# Characterizing GLP-1 Receptor Agonist Use in Preadolescent and Adolescent Populations

**DOI:** 10.1001/jamanetworkopen.2024.39887

**Published:** 2024-10-16

**Authors:** Margaret G. Miller, Pauline Terebuh, David C. Kaelber, Rong Xu, Pamela B. Davis

**Affiliations:** 1 Center for Artificial Intelligence in Drug Discovery, Case Western Reserve University School of Medicine, Cleveland, Ohio; 2 Departments of Medicine, Pediatrics, and Population and Quantitative Health Sciences and the Center for Clinical Informatics Research and Education, The MetroHealth System, Cleveland, Ohio; 3 Center for Community Health Integration, Case Western Reserve University School of Medicine, Cleveland, Ohio

## Abstract

This cross-sectional study examines the characteristics of preadolescent and adolescent populations who received glucagon-like peptide-1 (GLP-1) receptor agonists.

## Introduction

The first glucagon-like peptide-1 receptor agonists (GLP-1RAs) to be approved for preadolescent and adolescent populations was liraglutide in 2019, which treated type 2 diabetes (T2D). Exenatide and semaglutide approval for preadolescent and adolescent T2D and obesity soon followed.^1^ A recent study reported a 600% increase in patients aged 12 to 25 years who were prescribed GLP-1RAs between 2020 and 2023.^2^

Little is known about demographics or comorbidities of preadolescent and adolescent patients prescribed GLP-1RAs. We characterize patient demographics and prevalence of comorbid conditions that are approved indications for these drugs along with other common comorbidities in this population.

## Methods

This cross-sectional study followed the Strengthening the Reporting of Observational Studies in Epidemiology (STROBE) reporting guideline. The MetroHealth System institutional review board has deemed that studies that do not include human participants do not require approval. Data were obtained using the TriNetX Analytics Platform (US Collaborative Network) aggregating deidentified electronic health records (EHRs) of more than 110 million patients, which includes 64 large health care organizations across the country.

The study population included patients aged 10 to 17 years with a visit between 2019 and 2023 (when GLP1-RAs were approved for this age range)^1,3^ and first prescription for a GLP-1RA vs a date-matched visit with no prescription for a GLP-1RA. Patient demographics, comorbid conditions, body mass index (BMI), and prescriptions for other antidiabetic drugs prior to GLP-1RA prescription were compared with age-matched controls with supplemental analyses matching on all demographic characteristics. Race and ethnicity data were considered in this study because race and ethnicity are risk factors for T2D and obesity. Statistical analyses were conducted in the TriNetX Analytics Platform using the embedded statistical R version 3.2.3 (R Project for Statistical Computing). Data analyses were performed from February 24 to June 17, 2024. Statistical significance was set at *P* < .05, and tests were 2-sided. Further details are given in the eMethods in Supplement 1.

## Results

This study included 11 380 participants (6292 females [55.3%]; 4927 males [43.3%]; 2490 Black participants [21.9%]; 2373 Hispanic participants [20.9%]; 5467 White participants [48.0%]). The Figure summarizes overarching trends in GLP-1RA prescriptions among preadolescent and adolescent patients with visits at health care organizations contributing to TriNetX. From the study population of 7 055 472 preadolescent and adolescent patients, 5690 were prescribed a GLP-1RA. Compared with propensity-score age-matched controls, patients prescribed GLP-1RAs were more commonly female (3438 [60.4%] vs 2854 [50.2%]; *P* < .001), Black or African American individuals (1590 [27.9%] vs 900 [15.8%]; *P* < .001), and Hispanic or Latinx individuals (1441 [25.3%] vs 932 [16.4%]; *P* < .001), and less commonly Asian individuals (100 [1.8%] vs 204 [3.6%]; *P* < .001) or White individuals (22618 [46.0%] vs 2849 [50.1%]; *P* < .001).

**Figure.**
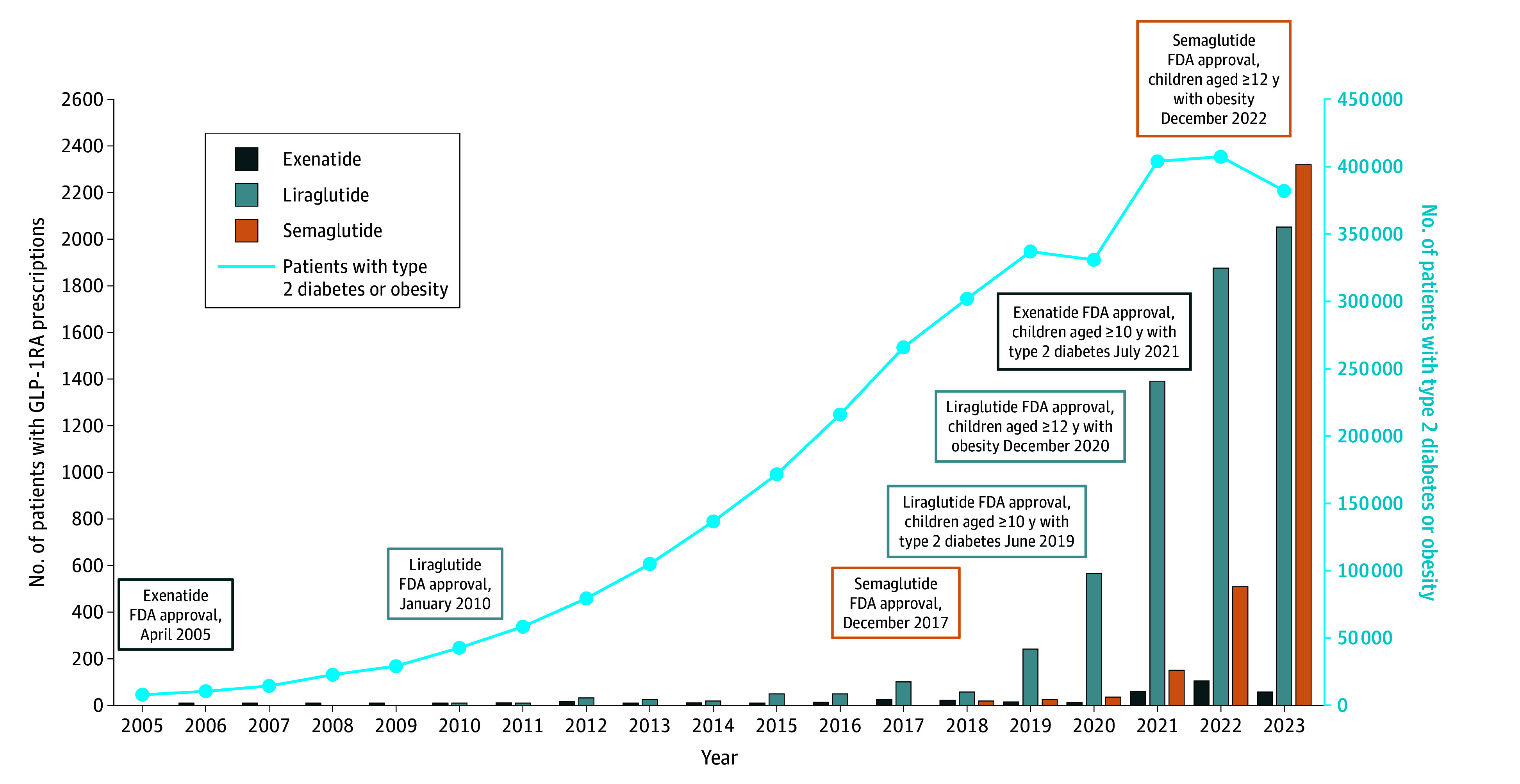
Overarching Glucagon-Like Peptide-1 Receptor Agonist (GLP-1RA) Prescriptions in Preadolescents and Adolescents GLP-1RA usage following US Food and Drug Administration (FDA) approval for exenatide, liraglutide, and semaglutide. The left ordinate demonstrates the number of patients aged 10 to 17 years who were prescribed GLP-1RAs at TriNetX health care organizations (bars). The right ordinate indicates the number of patients aged 10 to 17 years with a diagnosis of type 2 diabetes or obesity in each year at TriNetX health care organizations (blue line).

Preexisting *International Statistical Classification of Diseases and Related Health Problems, Tenth Revision *(*ICD-10*) encounter diagnosis codes of T2D (2629 [46.2%]), metabolic syndrome (912 [16.0%]), and type 1 diabetes (T1D) (1059 [18.6%]) were prevalent among patients receiving GLP-1RAs vs 10 patients (0.2%) with each condition in the control group, which were matched for age and demographics (Table). Among patients receiving a GL1-RA with BMI measurements within 2 years prior to starting the medication, 3371 patients (87.1%) had BMI in the 95th percentile or higher, compared with 1274 patients (32.9%) who did not receive GLP-1RAs. Prior to receiving a GLP-1RA prescription, 3755 patients (66%) had a prescription for insulin, metformin, or orlistat. When compared with the same diagnoses in matched controls, mood and anxiety disorders were more common in those with T2D (mood disorders: 331 [13.5%] vs 224 [9.1%]; *P* < .001; anxiety: 380 [15.5%] vs 268 [10.9%]; *P* < .001) and in those without T2D (mood disorders: 535 [19.7%] vs 95 [3.5%]; *P* < .001; anxiety: 697 [25.6%] vs 115 [4.2%]; *P* < .001) at the time GLP-1RAs were prescribed.

**Table.** Comparison of the Characteristics of Patients Aged 10 to 17 Years With First Prescription of GLP-1RAs Compared With Those With a Date-Matched Medical Visit and Never Prescribed GLP-1RAs

Characteristic^a^	Prescription (n = 5690)	No prescription (n = 5690)	*P* value^b^
**Comparison of demographics to adolescents with similar age** ^c^			
Age when data accessed, mean (SD), y^c^	16.7 (2.2)	16.7 (2.2)	NA
Age at index, mean (SD), y^c^	14.8 (1.8)	14.8 (1.8)	NA
Sex			
Female	3438 (60.4)	2854 (50.2)	<.001
Male	2204 (38.7)	2723 (47.9)	<.001
Unknown sex	48 (0.8)	113 (2.0)	<.001
Ethnicity^d^			
Hispanic or Latinx	1441 (25.3)	932 (16.4)	<.001
Not Hispanic or Latinx	3420 (60.1)	3085 (54.2)	<.001
Unknown	829 (14.6)	1673 (29.4)	<.001
Race^d^			
American Indian or Alaska Native	41 (0.7)	19 (0.3)	.004
Asian	100 (1.8)	204 (3.6)	<.001
Black or African American	1590 (27.9)	900 (15.8)	<.001
Native Hawaiian or other Pacific Islander	16 (0.3)	33 (0.6)	.02
White	2618 (46.0)	2849 (50.1)	<.001
Unknown	781 (13.7)	1267 (22.3)	<.001
Other race^e^	544 (9.6)	418 (7.4)	<.001
**Comparison of characteristics to adolescents with similar demographics** ^f^			
Diabetes			
Type 2 diabetes	2629 (46.2)	10 (0.2)^g^	<.001
Type 1 diabetes	1059 (18.6)	10 (0.2)^g^	<.001
Metabolic syndrome and other insulin resistance	912 (16.0)	10 (0.2)^g^	<.001
Hemoglobin A_1c_ measurement^h^	3801 (66.8)	67 (1.2)	NA
Mean (SD), %	7.2 (2.6)	5.6 (1.4)	<.001
Long-term (current) use of insulin	1181 (20.8)	10 (0.2)^g^	<.001
Metformin	3331 (58.5)	10 (0.2)^g^	<.001
Insulin	1848 (32.5)	10 (0.2)^g^	<.001
Orlistat	20 (0.4)	0	<.001
Obesity and overweight in previous 2 y			
Pediatric BMI, ≥95th percentile for age (n = 3872)^i^	3371 (87.1)	1274 (32.9)	<.001
Pediatric BMI, 85th to <95th percentile for age (n = 3872)^i^	126 (3.3)	500 (12.9)	<.001
Psychiatric diagnoses			
Anxiety, dissociative, stress-related, somatoform, and other nonpsychotic mental disorders	1413 (24.8)	103 (1.8)	<.001
Mood (affective) disorders	1051 (18.5)	47 (0.8)	<.001

Abbreviations: BMI, body mass index; GLP-1RA, glucagon-like peptide-1 receptor agonist; NA, not applicable.

To convert to proportion of total hemoglobin, multiply by 0.01.

^a^ Look-back period for characteristics was anytime to 1 day before the index event, unless otherwise noted.

^b^ An uncorrected *P* < .05 was used as the threshold for statistical significance.

^c^ Cohorts were matched on age only for comparison of demographic characteristics between cohorts with and without prescription.

^d^ Race and ethnicity were self-reported or clinician observed.

^e^ Includes a patient if they identify as multiracial (more than 1 race specified) or if the patient belongs to a race that is not listed in the aforementioned options.

^f^ Cohorts were matched on both age and demographics for comparison of prevalence of comorbidities between cohorts with and without prescription.

^g^ The TriNetX platform reports counts of 1 to 10 as 10 for addition privacy protection.

^h^ The most recent hemoglobin A_1c_ value prior to the index event. Not all patients in the prescription cohort had a value documented and few patients in the comparison cohort had a value documented.

^i^ A matched subcohort including only patients with a BMI measurement documented within 2 years prior to index event were compared.

## Discussion

In accordance with US Food and Drug Administration-approved indications and pediatric prescribing guidelines, EHR analysis showed that patients aged 10 to 17 years who were prescribed GLP-1RAs were more likely to have comorbid T2D, BMI in the 95th percentile or higher, and previous prescription of metformin.^4^ GLP-1RA use for those with a recorded T1D diagnosis is common.^5^ This study has limitations, including the lack of recorded indication for GLP-1RA administration, inability to confirm duration of GLP-1RA usage, and potential biases (eg, misclassification of diagnoses or overlapping diagnoses for a given patient) due to the retrospective analysis using EHRs.

A deep understanding of the long-term impacts (ie, physical, mental, sociocultural, etc) of GLP-1RAs on preadolescent and adolescent patients is crucial to better inform prescription practices and is an important direction for future studies. The demographic and comorbidity characteristics associated with current GLP-1RA prescription practices can help to contextualize the long-term impacts of these drugs.

## Notes

Supplement 1.
eMethods.

Supplement 2.
Data Sharing Statement

## References

[1] Yan Y, Gong Y, Jiang M, . Utilization of glucagon-like peptide-1 receptor agonists in children and adolescents in China: a real-world study. Front Endocrinol (Lausanne). 2023;14:1170127. doi:10.3389/fendo.2023.1170127 37383395 PMC10293789

[2] Lee JM, Sharifi M, Oshman L, Griauzde DH, Chua KP. Dispensing of glucagon-like peptide-1 receptor agonists to adolescents and young adults, 2020-2023. JAMA. 2024;331(23):2041-2043. doi:10.1001/jama.2024.7112 38776113 PMC11112492

[3] Alorfi NM, Alshehri FS. Usage of glucagon-like peptide-1 for obesity in children; updated review of clinicaltrials.gov. J Multidiscip Healthc. 2023;16:2179-2187. doi:10.2147/JMDH.S419245 37547806 PMC10402718

[4] Hampl SE, Hassink SG, Skinner AC, . Clinical practice guideline for the evaluation and treatment of children and adolescents with obesity. Pediatrics. 2023;151(2):e2022060640. doi:10.1542/peds.2022-060640 36622115

[5] Guyton J, Jeon M, Brooks A. Glucagon-like peptide 1 receptor agonists in type 1 diabetes mellitus. Am J Health Syst Pharm. 2019;76(21):1739-1748. doi:10.1093/ajhp/zxz179 31612934

