# Supplementary material for: Characterizing GLP-1 Receptor Agonist Use in Preadolescent and Adolescent Populations
Source: JAMA Netw Open. 2024 Oct 16;7(10):e2439887. doi: 10.1001/jamanetworkopen.2024.39887 (PMC11581558; doi:10.1001/jamanetworkopen.2024.39887)
Supplement: Supplement 1. — eMethods. [file jamanetwopen-e2439887-s001.pdf]

Supplemental Online Content

Miller MG, Terebuh P, Kaelber DC, Xu R, Davis PB. Characterizing GLP-1 receptor agonist use in preadolescent and adolescent populations. *JAMA Netw Open*. 2024;7(10):e2439887. doi:10.1001/jamanetworkopen.2024.39887

eMethods.

This supplemental material has been provided by the authors to give readers additional information about their work.

© 2024 Miller MG et al. *JAMA Network Open.*

eMethods

The data used in this study were accessed from May to June of 2024 from the TriNetX US Collaborative Network without the use of natural language processing. This resource provides access to electronic health records (diagnoses, procedures, medications, laboratory values, genomic information) from over 100 million patients from 64 healthcare organizations, which is de-identified per criteria from the Health Insurance Portability and Accountability Act (HIPAA), Section §164.514(a) of the HIPAA Privacy Rule. Some HCOs that contribute to the network date-shift individual EHRs from 1 to 365 days on the calendar as an additional privacy protection, however the relative timing within each health record is maintained. The MetroHealth System in Cleveland, Ohio, IRB has determined that research using TriNetX in ways such as described in this manuscript is not Human Subject Research and therefore exempt from IRB review.

The TriNetX platform de-identifies and aggregates electronic health record (EHR) data from 64 contributing healthcare systems, most of which are large academic medical institutions with both inpatient and outpatient facilities at multiple locations, across all 50 states in the US. Patient EHR data includes information from hospitals, primary care, and specialty treatment providers, covering diverse geographic locations, age groups, racial and ethnic groups, income levels and insurance types including various commercial insurances, governmental insurance (Medicare and Medicaid), self-pay/uninsured, worker compensation insurance, and military/VA insurance among others. Race and ethnicity data in TriNetX is derived from self-reports or provider observations in the clinical EHR systems, which is then mapped to the following categories: (1) Race: Asian, American Indian or Alaskan Native, Black or African American, Native Hawaiian or Other Pacific Islander, White, Unknown race; and (2) Ethnicity: Hispanic or Latino, Not Hispanic or Latino, Unknown Ethnicity.

The lookback period for patient demographics, comorbid conditions, and prescriptions for other diabetic drugs was anytime up to 1 day before the index event: the first GLP-1RA prescription (exposure group) or patients who had a visit documented and no prescription for GLP-1RAs (control group). The data presented in Table 1 represent the most recently recorded information in a patient's chart. The lookback period for the sub analysis of patient BMI data was within 2 years prior to the index event.

GLP-1RA Prescription Cohort: Inclusion Criteria Codes

Visit – 10-17 years (Included if occurred between January 1, 2019 and December 31, 2023)
RxNorm 1991302: Semaglutide – 10-17 years (Included if occurred between January 1, 2022 and December 31, 2023)
RxNorm 475968: Liraglutide – 10-17 years (Included if occurred between January 1, 2019 and December 31, 2023)
RxNorm 60548: Exenatide – 10-17 years (Included if occurred between January 1, 2021 and December 31, 2023)

No GLP-1RA Prescription Cohort: Inclusion and Exclusion Criteria Codes

© 2024 Miller MG et al. *JAMA Network Open.*

Included if the code below occurred between January 1, 2019 and December 31, 2023:

Visit – 10-17 years

Excluded if any of the codes below occurred between January 1, 2019 and December 31, 2023:

RxNorm 1991302: Semaglutide
RxNorm 475968: Liraglutide
RxNorm 60548: Exenatide

GLP-1RA Prescription Cohort with BMI recorded: Inclusion Criteria Codes

Visit – 10-17 years (Included if occurred between January 1, 2019 and December 31, 2023)
RxNorm 1991302: Semaglutide – 10-17 years (Included if occurred between January 1, 2022 and December 31, 2023)
RxNorm 475968: Liraglutide – 10-17 years (Included if occurred between January 1, 2019 and December 31, 2023)
RxNorm 60548: Exenatide – 10-17 years (Included if occurred between January 1, 2021 and December 31, 2023)
Z68.5: Body mass index [BMI] pediatric

No GLP-1RA Prescription Cohort with BMI recorded: Inclusion and Exclusion Criteria Codes

Included if the code below occurred:

Visit – 10-17 years (between January 1, 2019 and December 31, 2023)
Z68.5: Body mass index [BMI] pediatric

Excluded if any of the codes below occurred between January 1, 2019 and December 31, 2023:

RxNorm 1991302: Semaglutide
RxNorm 475968: Liraglutide
RxNorm 60548: Exenatide

Stratified Analysis for Mood and Anxiety Disorders: T2DM with GLP-1RA Cohort Inclusion Criteria Codes

Included if the code below occurred:

Visit – 10-17 years (between January 1, 2019 and December 31, 2023)

To be included, any one of the following codes must have also occurred between January 1, 2019 and December 31, 2023:

RxNorm 1991302: Semaglutide – 10-17 years

© 2024 Miller MG et al. *JAMA Network Open.*

RxNorm 475968: Liraglutide – 10-17 years
RxNorm 60548: Exenatide – 10-17 years

To be included, any one of the following codes must have also occurred between January 1, 2019 and December 31, 2023:

E10: Type 1 diabetes mellitus
E11: Type 2 diabetes mellitus

Stratified Analysis for Mood and Anxiety Disorders: T2DM with No GLP-1RA Cohort Inclusion and Exclusion Criteria Codes

Included if the code below occurred:

Visit – 10-17 years (between January 1, 2019 and December 31, 2023)

To be included, any one of the following codes must have also occurred between January 1, 2019 and December 31, 2023:

E10: Type 1 diabetes mellitus
E11: Type 2 diabetes mellitus

Excluded if any of the codes below occurred at any time:

RxNorm 1991302: Semaglutide
RxNorm 475968: Liraglutide
RxNorm 60548: Exenatide

Stratified Analysis for Mood and Anxiety Disorders: No T2DM with GLP-1RA Cohort Inclusion and Exclusion Criteria Codes

Included if the code below occurred:

Visit – 10-17 years (between January 1, 2019 and December 31, 2023)

To be included, any one of the following codes must have also occurred between January 1, 2019 and December 31, 2023:

RxNorm 1991302: Semaglutide – 10-17 years
RxNorm 475968: Liraglutide – 10-17 years
RxNorm 60548: Exenatide – 10-17 years

Excluded if any of the codes below occurred at any time:

E10: Type 1 diabetes mellitus
E11: Type 2 diabetes mellitus

© 2024 Miller MG et al. *JAMA Network Open.*

Stratified Analysis for Mood and Anxiety Disorders: No T2DM with No GLP-1RA Cohort Inclusion and Exclusion Criteria Codes

Included if the code below occurred:

Visit – 10-17 years (between January 1, 2019 and December 31, 2023)

Excluded if any of the codes below occurred at any time:

E10: Type 1 diabetes mellitus
E11: Type 2 diabetes mellitus
RxNorm 1991302: Semaglutide
RxNorm 475968: Liraglutide
RxNorm 60548: Exenatide

© 2024 Miller MG et al. *JAMA Network Open.*
