# Supplementary material for: Characterizing GLP-1 Receptor Agonist Use in Preadolescent and Adolescent Populations
Source: JAMA Netw Open. 2024 Oct 16;7(10):e2439887. doi: 10.1001/jamanetworkopen.2024.39887 (PMC11581558; doi:10.1001/jamanetworkopen.2024.39887)
Supplement: Supplement 2. — Data Sharing Statement [file jamanetwopen-e2439887-s002.pdf]

Data Sharing Statement

Miller. Characterizing GLP-1 Receptor Agonist Use in Preadolescent and Adolescent Populations. *JAMA Netw Open*. Published October 16, 2024.
doi:10.1001/jamanetworkopen.2024.39887

Data
Data available: No

Additional Information
Explanation for why data not available: This study used population-level aggregate and HIPAA de-identified data collected by the TriNetX platform and available from TriNetX, LLC (https://trinetx.com/), but third-party restrictions apply to the availability of these data. The data were used under license for this study with restrictions that do not allow for the data to be redistributed or made publicly available. To gain access to the data, a request can be made to TriNetX (join@trinetx.com), but costs may be incurred, and a data-sharing agreement may be necessary. Data specific to this study including diagnosis codes and cohort characteristics in aggregated format are included in the manuscript as tables, figures, and supplementary files. Data through the TriNetX platform is queried in real-time with results being returned typically in seconds to minutes. Data from the underlaying electronic health records of participating healthcare organizations is refreshed in the TriNetX platform from daily to every couple of months depending on the healthcare organization.
